# Multi-targeted Antisense Oligonucleotide Delivery by a Framework Nucleic Acid for Inhibiting Biofilm Formation and Virulence

**DOI:** 10.1007/s40820-020-0409-3

**Published:** 2020-03-17

**Authors:** Yuxin Zhang, Xueping Xie, Wenjuan Ma, Yuxi Zhan, Chenchen Mao, Xiaoru Shao, Yunfeng Lin

**Affiliations:** State Key Laboratory of Oral Diseases, National Clinical Research Center for Oral Diseases, West China Hospital of Stomatology, Sichuan University, Chengdu, 610041 People’s Republic of China

**Keywords:** Biofilm, Framework nucleic acid, Multi-targeting, Antisense oligonucleotide, Delivery system

## Abstract

Biofilm formation is responsible for numerous chronic infections and represents a serious health challenge. Bacteria and the extracellular polysaccharides (EPS) cause biofilms to become adherent, toxic, resistant to antibiotics, and ultimately difficult to remove. Inhibition of EPS synthesis can prevent the formation of bacterial biofilms, reduce their robustness, and promote removal. Here, we have developed a framework nucleic acid delivery system with a tetrahedral configuration. It can easily access bacterial cells and functions by delivering antisense oligonucleotides that target specific genes. We designed antisense oligonucleotide sequences with multiple targets based on conserved regions of the VicK protein-binding site. Once delivered to bacterial cells, they significantly decreased EPS synthesis and biofilm thickness. Compared to existing approaches, this system is highly efficacious because it simultaneously reduces the expression of all targeted genes (*gtfBCD*, *gbpB*, *ftf*). We demonstrate a novel nucleic acid-based nanomaterial with multi-targeted inhibition that has great potential for the treatment of chronic infections caused by biofilms.

## Highlights

- A framework nucleic acid delivery system was developed through self-assembly, which can deliver antisense oligonucleotides against multiple targets in bacterial cells.
- The ASOs-tFNAs (750 nM) was found to simultaneously inhibit the expression of *gtfBCD*, *gbpB*, and *ftf*, and significantly reduce the extracellular polysaccharide synthesis and biofilm thickness.

## Graphical abstract

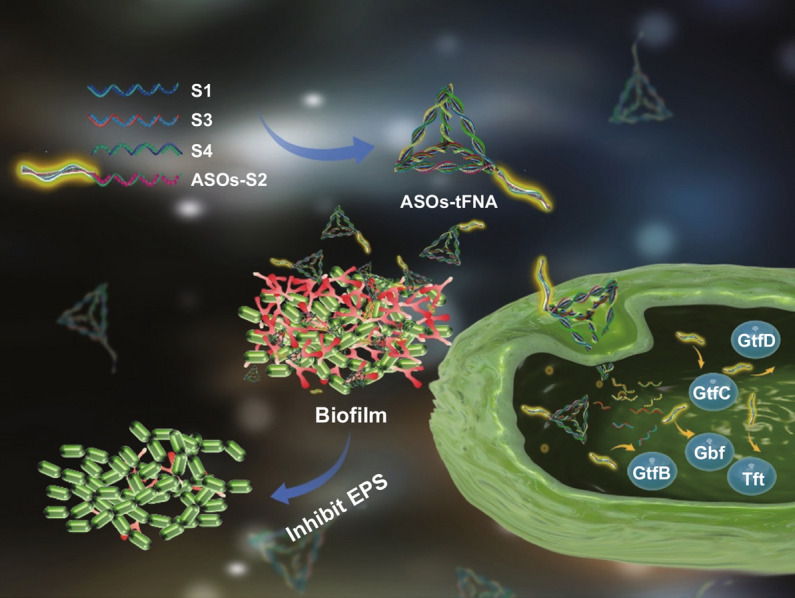

## Introduction

Biofilms represent structured bacterial communities attached to an extracellular matrix secreted by the bacteria on the surface of a living or inanimate body [1]. Bacteria are embedded in these extracellular matrices to protect themselves. Biofilms contain various biological macromolecules, such as extracellular polysaccharides (EPS) and nucleic acids. The EPS matrix enhances the adhesion of bacterial cells and promotes surface microbial accumulation and cohesion, condensing dense cell aggregates and resulting in extremely structured and adherent bacterial biofilms [2]. Therefore, bacteria that form biofilms are 500 to 5000 times more resistant to antibiotics than planktonic cells, including those that are normally sensitive to antibiotics [3, 4]. There are three main reasons for such high drug resistance. First, the metabolic rate of bacteria proliferating within biofilms decreases due to competition for nutrients and space, which makes them less susceptible to growth-targeting antibiotic action [5]. Second, the protein and polysaccharide components in the EPS matrix can prevent or delay the penetration of antibiotics into biofilms, giving mature cells located deep in the matrix more time to develop resistance [5, 6]. Third, individual-resistant bacteria release antibiotic resistance factors, enabling the entire biofilm community to become resistant through a process known as passive resistance [7]. Resistance-related genes can be shared between different strains in biofilms by lateral gene transfer. These factors are also the main mechanism behind chronic infections, which constitute 60–80% of human infectious diseases. Microbial biofilm formation is regarded as a major virulence factor for local chronic infections of the heart, lung tissue, skin, and oral cavity [1]. Together with aging of the population, diabetes, and the prevalence of obesity [8], chronic infections have become a major public health issue. Consequently, addressing biofilm infection has become a key area of research direction. Given that traditional treatments, such as mechanical debridement, antibiotics, or biofilm destroyers, are insufficient against the biofilm’s self-protecting ability and strong toxicity, the only viable option is to prevent the early stages of biofilm formation [9, 10]. Biofilm formation is a dynamic process, in which bacteria secrete large amounts of extracellular polysaccharides during the early colonization and aggregation steps. Therefore, EPS-related genes and proteins have become important targets for early intervention in biofilm formation [11].

Existing methods for combating biofilm infection include nanocoatings that inhibit adhesion, a combination of mechanical debridement and antibiotics, and liposomal nanoparticles for drug delivery [1, 6]. The recent rapid development of nanomaterials has sparked therapeutic strategies that selectively target biofilm matrix components or that introduce bacterial-specific ligands to increase the specificity of nanoparticles, thus improving efficacy and biocompatibility [12–14]. However, presently, the nanoparticles used are mostly liposomes or gold nanoparticles. These nanoparticles have high cytotoxicity, affect the normal physiological activities of host cells, have low biocompatibility, and low editability [15, 16]. Recently, a framework nucleic acid (FNA) with a tetrahedral conformation has been confirmed to possess anti-inflammatory and anti-oxidative properties and promote proliferation and differentiation of animal cells and the capacity to enter bacterial cells [16–18]. In particular, FNAs are highly editable and can function as carriers for nucleic acid drugs to enter cells [18–20]. Indeed, various such drugs, including aptamers or cytosine-phosphate-guanine, have been designed for FNAs to provide anticancer or immunostimulatory activities [21–23].

Based on these factors, we have developed a new drug delivery system that targets biofilms. We designed a multi-targeting antisense nucleotide sequence to be carried by FNAs into bacterial biofilms. The phosphorothioated DNA and 2′ O-Me RNA on the sequence provided additional stability and increased its affinity for the target sequences [24, 25]. In this study, we selected the *Streptococcus mutans* biofilm, which is related to the occurrence of various oral diseases [26]. Secretion of EPS in the early stages of *S. mutans* biofilm formation is regulated mainly by the glucosyltransferase gene family (*gtfB, gtfC, gtfD*) and the glucan-binding protein *gbpB* gene [27]. Therefore, we targeted these genes. Senadheera et al. demonstrated that a VicRK signal transduction system in *S. mutans* biofilm affected *gtfBCD, gbpB*, and *ftf* (encoding an adhesion-associated protein) expression, biofilm formation, and genetic development [28–31]. This occurs because the VicK protein binds to the highly conserved promoter region of the *gtfBCD, gbpB,* and *ftf* genes [27, 32, 33]. Accordingly, the antisense complex sequence used here was based on this conserved sequence. We then proceeded with the construction and characterization of this novel nucleic acid drug delivery system. The system proved to be more efficient and smarter than our previous design, achieving good inhibition during early formation of *S. mutans* biofilms, caused by reduced expression of the target genes and blocking of EPS production. To the best of our knowledge, this is the first report of FNAs acting as a drug delivery vehicle that successfully inhibits the formation of bacterial biofilms. It also confirms the vast potential of the system for the treatment of bacterial infections.

## Experimental

### Multi-targeted Antisense Oligonucleotide Design

VicK proteins bind specifically to the *gtfBCD*, *gbpB*, and *ftf* promoter regions [30, 32]. Dubrace et al. determined the VicK binding consensus sequence to be TGTWAHNNNNNTGTWAH (where W is A or T and H is A, T, or C) [34]. Based on this information, we designed the antisense oligonucleotide sequence DTWACANNNNNDTAACA (where D is A, G, or T and W is A or T) to target the conserved sequences of the gtfBCD, gbpB, and ftf promoter regions [32]. Because pure DNA or RNA is susceptible to nuclease degradation, we designed a sequence of nested antisense oligonucleotides made of DNA and RNA, with DNA modified by phosphorothioate and RNA by 2′ O-Me [35–37].

### Fabrication and Characterization

The tFNA was self-assembled from four different ssDNAs (denoted as S1, S2, S3, and S4) in accordance with previous studies (Table 1). Multi-targeted antisense oligonucleotides (ASOs) were linked to the 5′ of the S2 ssDNA to produce an ASOs-tetrahedral FNA (tFNA) delivery system [12, 37, 38]. Briefly, ASOs-tFNAs were assembled using an equimolar ratio of S1, ASOs-S2, S3, and S4. They were mixed in TM buffer, heated to 95 °C for 10 min, and cooled quickly to 4 °C for 20 min [39, 40]. Finally, the ASOs-tFNA complex was purified by high-performance liquid chromatography [41].

**Table 1:** Base sequence of each ssDNA

ssDNA	Base sequence	Direction
Cy5-S1	Cy5-ATTTATCACCCGCCATAGTAGACGTATCACCAGGCAGTTGAGACGAACATTCCTAAGTCTGAA	5′ → 3′
S1	ATTTATCACCCGCCATAGTAGACGTATCACCAGGCAGTTGAGACGAACATTCCTAAGTCTGAA	5′ → 3′
S2	ACATGCGAGGGTCCAATACCGACGATTACAGCTTGCTACACGATTCAGACTTAGGAATGTTCG	5′ → 3′
S3	ACTACTATGGCGGGTGATAAAACGTGTAGCAAGCTGTAATCGACGGGAAGAGCATGCCCATCC	5′ → 3′
S4	ACGGTATTGGACCCTCGCATGACTCAACTGCCTGGTGATACGAGGATGGGCATGCTCTTCCCG	5′ → 3′
ASO	DTWACANNNNNDTAACA	5′ → 3′

To prove the successful synthesis of ASOs-tFNA, gel electrophoresis, atomic force microscopy (AFM) (DI Multimode-VIII; Bruker Nano Inc., Billerica, MA, USA), and transmission electron microscopy (TEM) (Tecnai G2 F20 S-TWIN; FEI, Hillsboro, OR, USA) were used to analyze its structure [20]. An 8% non-denaturing polyacrylamide gel electrophoresis in 1 × TAE buffer was run at 4 °C for about 1.5 h to measure the relative molecular weights of each ssDNA and two strands of ssDNA, as well as combinations of three ssDNAs and ASOs-tFNA. The morphology and approximate sizes of pure tFNA and ASOs-tFNA were confirmed by AFM and TEM. Briefly, 10 μL of ASOs-tFNA was dropped onto freshly cleaved mica flakes, dried for 15 min, and measured by AFM [42]. Similarly, 5 μL of ASOs-tFNA was dripped on copper grids and the sample was dried under infrared radiation for 20 min prior to TEM observation. Then, hydrodynamic sizes and zeta potentials were analyzed with a Zetasizer Nano ZS instrument (Malvern Instrument Ltd., Malvern, UK). Each measurement was repeated three times [43].

### Nucleic Acid Strands, Test Bacteria, and Growth Conditions

Nucleic acid strands used in the experiments were synthesized and purified by TaKaRa (Dalian, China). *S. mutans* UA159 was commercially obtained from the American Type Culture Collection (Manassas, VA, USA). The bacteria were grown on brain heart infusion (BHI) medium (Difco, Sparks, MD, USA) in an Incubator (Thermo Fisher Scientific, Waltham, MA, USA) at 37 °C and 5% CO_2_. For all the experiments, bacteria were diluted to 1 × 10^6^ CFU mL^−1^ in BHI medium. For bacterial biofilm growth, 1% sucrose (Sigma, St. Louis, MO, USA) was added to BHI medium (BHIS). To ensure TM buffer had no impact on the experimental results; control groups and experimental groups contained the same concentration of TM buffer in each assay.

### Uptake of FNA, ASOs, and ASOs-tFNAs by Bacteria

To verify that ASOs-tFNAs successfully penetrated *S. mutans* biofilms, Cyanine-5 (Cy5)-tFNAs (500 or 750 nM), Cy5-ASOs (500 or 750 nM), and Cy5-ASOs-tFNAs (500 or 750 nM) were used to treat bacteria for 12 h on BHI medium without sucrose. The strains were collected and washed three times with PBS (15,000 rpm, 5 min). Each sample was resuspended in PBS in flow tubes, and each of these was subjected to flow cytometric analysis (FC500; Beckman-Coulter, Indianapolis, IN, USA) [12].

### Planktonic Bacteria Growth Assay

The strains were grown from a single colony in BHI both until early log phase was achieved at an optical density at 595 nm (OD595) of 0.2–0.3. After adjusting OD to a common value, bacteria were cultured in the presence of different concentrations of tFNAs and ASOs-tFNAs in BHI medium in a cell culture plate. An automated spectrophotometer (BioTek, Winooski, VT, USA) was used to measure planktonic bacterial growth, and OD595 readings were taken over a period of 24 h every 2 h, with plate shaking every 30 min.

### Biofilms Formation Assay

To test the inhibition of ASOs-tFNAs on biofilm formation, the experimental strains were inoculated into wells of a 96-well plate containing BHIS. Bacteria were treated with tFNAs, ASOs, or ASOs-tFNAs (500 or 750 nM) for 24 and 48 h. Then, culture medium was removed out and cells were washed twice with PBS. Cells were fixed by adding 100 μL methanol into each well and incubating for 15 min; after that, excess liquid was removed out and the wells were dried naturally. Next, 0.1% crystal violet staining solution was added to each well, and the plates were placed at 20 °C for 5 min [44]. After the liquid was removed from the wells, the plates were dried in a drying oven. Finally, 100 µL acetic acid (33%) was added to each well and incubated at 37 °C for 30 min to dissolve the crystal violet solution. OD595 of the samples was taken with a microplate reader [44].

### Microscopic Analysis of Bacterial Biofilms

An overnight culture of *S. mutans* was inoculated into BHIS with tFNA, ASOs, and ASOs-tFNAs (500 or 750 nM) and incubated on confocal plates for 24 h. We observed the architecture of bacterial biofilms by in situ labeling of *S. mutans* and EPS [45]. Alexa Fluor 647-labeled dextran conjugate (1 µM; Thermo Fisher Scientific) was added to BHIS medium before inoculation, and after incubation for 24 h [46], the medium was removed and each sample was washed twice with sterile PBS to remove planktonic and loosely bound cells. Next, SYTO™ 9 dye (Thermo Fisher Scientific) was added at a 100:1 ratio to label the biofilms for 15 min. The architecture of bacterial biofilms was examined by confocal laser scanning microscopy (A1R MP+; Nikon, Tokyo, Japan). We used Z sections to record the thickness of the biofilms, at 1-µm intervals. All samples were scanned at five randomly selected positions [47]. Finally, the confocal slices were reconstructed into three-dimensional images of the biofilms. We used COMSTAT image processing software to analyze the confocal image stacks and to calculate the biomass of EPS and bacterial cells [48].

### Scanning Electron Microscopy (SEM)

The impact of ASOs-tFNAs on biofilm structure and the amount of EPS was observed by SEM (FEI). An overnight culture of *S. mutans* was inoculated into a cell culture plate with glass coverslips, and BHIS medium was added. After 1 day of incubation, samples were washed in sterile PBS to remove planktonic bacteria and loosely adherent cells. Each sample was fixed with 2.5% glutaraldehyde at 4 °C overnight. Then, samples were washed once with sterile PBS and dehydrated in an absolute ethanol series to maintain the morphology of bacterial cells. Each sample was coated with gold and observed by SEM.

### Quantitative RT-PCR

Quantitative RT-PCR was used to verify the multiple targeting of ASOs-tFNAs. The expression of targeted genes was quantified by using 16S rRNA as a control gene [49]. The strains were grown in BHIS with tFNAs and ASOs-tFNAs (750 nM) to late logarithmic phase. Then, the strains were harvested by centrifugation (4000 × g, 4 °C, 10 min) and snap-frozen in TRIzol reagent (Thermo Fisher Scientific) until further use. Total RNA from each sample was extracted and purified with the RNeasy Mini Kit (Qiagen, Hilden, Germany) using a genomic DNA eliminator [12, 50]. The extracted RNA was dissolved in RNase-free water, and cDNA was prepared using a cDNA synthesis kit (TaKaRa, Dalian, China). Amplifications of all target mRNAs were performed by quantitative RT-PCR. The corresponding primer sets are listed in Table 2. The Livak method is used to calculate the relative expression of the target genes. In this experiment, the control group contained tFNAs [40].

**Table 2:** Primer sequences of relevant genes designed for quantitative PCR

mRNA	Primer pairs
16S RNA	Forward 5′-TCGTGTCGTGAGATGTTGGG-3′
	Reverse 5′-GTTTGTCACCGGCAGTCAAC-3′
*gtfB*	Forward 5′-CACTATCGGCGGTTACGAAT-3′
	Reverse 5′-CAATTTGGAGCAAGTCAGCA-3′
*gtfC*	Forward 5′-GATGCTGCAAACTTCGAACA-3′
	Reverse 5′-TATTGACGCTGCGTTTCTTG-3′
*gtfD*	Forward 5′-TTGACGGTGTTCGTGTTGAT-3′
	Reverse 5′-AAAGCGATAGGCGCAGTTTA-3′
*gbpB*	Forward 5′-ACAGCAACAGAAGCACAACCATC-3′
	Reverse 5′-CCACCATTACCCCAGTAGTTTCC-3′
*ftf*	Forward 5′-ATTGGCGAACGGCGACTTACTC-3′
	Reverse 5′-CCTGCGACTTCATTACGATTGGTC-3′

### Statistical Analysis

We performed all experiments at least in quadruplicate and reproduced three separate times. Statistical analyses of the results were performed with Prism 6 (GraphPad software Inc., San Diego, CA, USA) by one-way ANOVA. *P < 0.05, **P < 0.01, and ***P < 0.001 indicated statistically significant differences.

## Results and Discussion

### Characterization of tFNAs

**Fig. 1:**
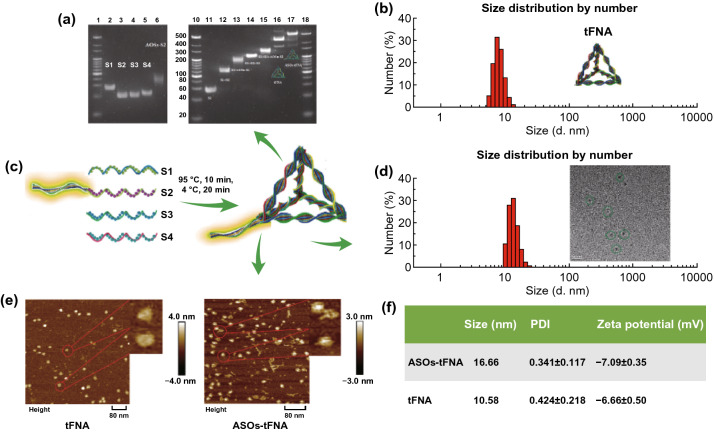
Characterization of tFNAs and ASOs-tFNAs. **a** Relative size of ssDNA (S1 to S4), partially assembled delivery systems, tFNA, and ASOs-tFNA based on non-denaturing polyacrylamide gel electrophoresis. Lanes 2 and 11: S1; lane 3: S2; lane 4: S3; lane 5: S4; lane 6: ASOs-S2; lane 12: S1 + S2; lane 13: S1 + ASOs-S2; lane 14: S1 + S2 + S3; lane 15: S1 + S3 + ASOs-S2; lane 16: tFNA; lane 17: ASOs-tFNA; lanes 1, 10, and 18: MW marker, **b** dynamic light scattering measurement of tFNA size distribution, **c** schematic representation of the synthesis of the tetrahedral FNA delivery system (ASOs-tFNA), **d** dynamic light scattering measurement of ASOs-tFNA size distribution and TEM image of ASOs-tFNAs (green dotted line), **e** AFM images of tFNA (red dotted line) and ASOs-tFNA (red dotted line), **f** summary of different parameters describing ASOs-tFNA and tFNA. (Color figure online)

The tFNAs were self-assembled from four different ssDNAs. Each ssDNA had 63 bases and formed one of the four faces of the tFNA by binding to the corresponding region of the other chains [12, 38]. The tFNA carrying ASOs (ASOs-tFNA) was constructed by linking ASOs to the 5′ of S2 ssDNA (Fig. 1c). The successful self-assembly of ASOs-tFNA was verified by non-denaturing polyacrylamide gel electrophoresis (Fig. 1a) and confirmed by the slower migration of ASOs-tFNA compared to ssDNA (lanes 2–5 and 11 in) or a combination of two (lanes 12 and 13), three (lanes 14 and 15), or four strands (lanes 16 and 17). The morphology of tFNA and ASOs-tFNA was characterized by AFM (Fig. 1e), and an approximate size of 10 nm was calculated for the ASOs-tFNA monomer. As shown in Fig. 1d, the hydrodynamic size of ASOs-tFNA was 16.66 nm, whereas that of tFNA alone was 10.58 nm. The size difference further proved the successful construction of ASOs-tFNA. There was, however, no significant difference in zeta potentials (Fig. 1f), indicating that the ASOs-tFNA delivery system was as stable as tFNA alone.

### ASO-tFNAs Taken Up by *S. mutans*

**Fig. 2:**
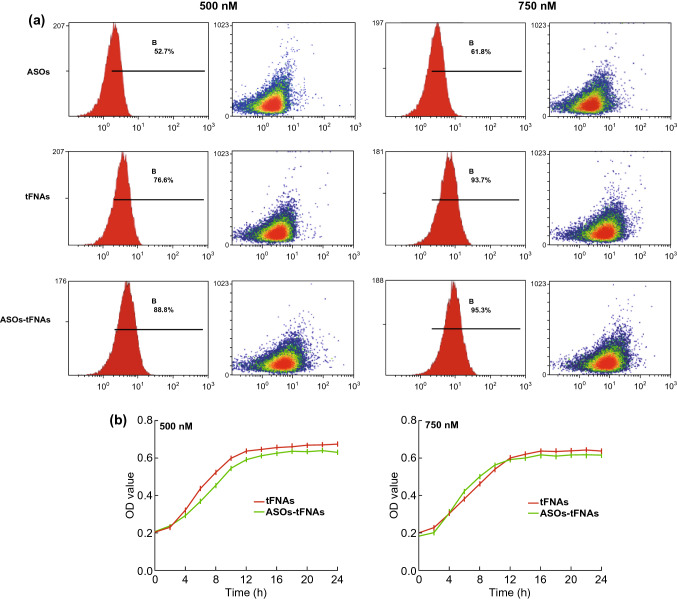
ASOs-tFNAs are successfully taken up by *S. mutans* biofilms. **a** Flow cytometry analysis of the uptake rates by *S. mutans* incubated in the presence of different concentrations of Cy5-labeled ASOs, tFNAs, and ASOs-tFNA for 12 h, **b** effect of tFNAs and ASOs-tFNAs on growth of planktonic *S. mutans* cells at different concentrations of tFNAs or ASOs-tFNAs. Values represent the mean ± SD (*n* = 3)

To induce any effect on bacterial biofilm formation and virulence, it was first necessary to ascertain the ingestion of ASOs-tFNAs by *S. mutans*. Figure 2a presents the uptake rates of *S. mutans* incubated with different concentrations (500 or 750 nM) of Cy5-labeled tFNAs, ASOs, and ASOs-tFNAs over a period of 12 h as determined by flow cytometry. Accordingly, ASOs-tFNAs were successfully taken up by *S. mutans* in a dose-dependent manner and more efficiently than either tFNAs or ASOs alone.

### Effect of tFNAs Carrying Multi-targeting ASOs on Cell Growth

The genes targeted by the ASOs-tFNA delivery system, *gtfBCD, gbpB,* and *ftf*, will not expressed in planktonic Streptococcus and, therefore, were not expected to affect the performance of *S. mutans* in sucrose-free BHI medium. Indeed, as shown in Fig. 2b, ASOs-tFNAs did not significantly alter the growth curve of planktonic *S. mutans* cells.

### ASOs-tFNAs Prevent Bacterial Biofilm Formation

**Fig. 3:**
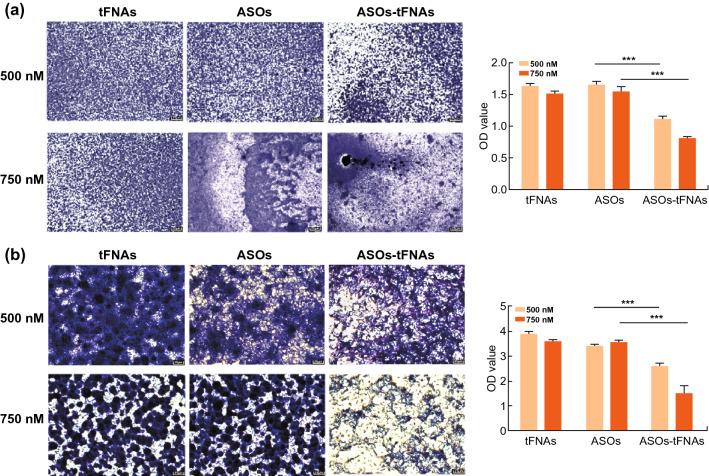
ASOs-tFNAs inhibit biofilm formation. **a** Crystal violet staining of *S. mutans* cells treated with different concentrations of tFNAs, ASOs, and ASOs-tFNAs for 24 h and semiquantitative analysis of biofilm formation, **b** crystal violet staining of *S. mutans* cells treated with different concentrations of tFNAs, ASOs, and ASOs-tFNAs for 48 h and semiquantitative analysis of biofilm formation. Data represent the mean ± SD (*n* = 3), ***P < 0.001. (Color figure online)

Crystal violet staining was used to study the effect of ASO-tFNAs on biofilm formation and quantitatively calculate the ability of bacteria to form biofilms at different time points. As shown in Fig. 3a, crystal violet staining gave a much darker and denser signal in tFNAs- and ASOs-treated cultures than in the ASOs-tFNAs sample, indicating that the biofilm was more mature. Further, the OD of the eluate was measured and ASOs-tFNAs inhibition of biofilm formation was confirmed. Figure 3b shows the biofilms treated with tFNAs, ASOs, and ASOs-tFNAs for a period of 48 h, with all images acquired at the same light intensity and magnification. Accordingly, mature biofilms formed following ASOs-tFNAs treatment were significantly less extensive than those of the control groups.

**Fig. 4:**
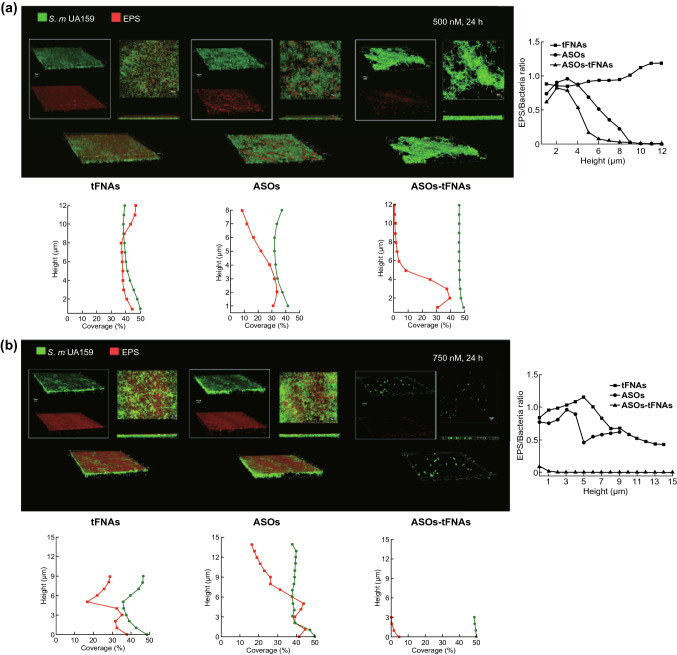
ASOs-tFNAs negatively affect the synthesis of EPS in *S. mutans* biofilms. **a** Dual-label imaging and three-dimensional visualization of EPS (red) and bacteria (green) in *S. mutans* biofilms after treatment with ASOs-tFNA at 500 nM, **b** dual-label imaging and three-dimensional visualization of EPS (red) and bacteria (green) in *S. mutans* biofilms after treatment with ASOs-tFNA at 750 nM. Quantitative analysis showing the distribution of EPS and bacteria from the glass surface to the liquid phase interface. Images are taken at × 100 magnification. (Color figure online)

To verify whether the inhibitory action of ASOs-tFNA on biofilm formation (Fig. 3) relied on inhibiting EPS synthesis, we evaluated the extent of bacterial growth and EPS accumulation by confocal microscopy. Figure 4 presents representative three-dimensional images of bacterial cells (green) and EPS (red), showing the biofilms’ morphology. The vertical distribution of bacteria and EPS from the surface of the glass to the liquid interface was quantified in COMSTAT using the confocal imaging data sets [51]. The results of data analysis indicated that ASOs-tFNAs disrupted the cells’ ability to synthesize EPS and reduced biofilm thickness. Analysis of the distribution of bacteria and EPS in the presence of different concentrations of ASOs-tFNAs (500 or 750 nM) further supported these conclusions (Fig. 4a, b).

**Fig. 5:**
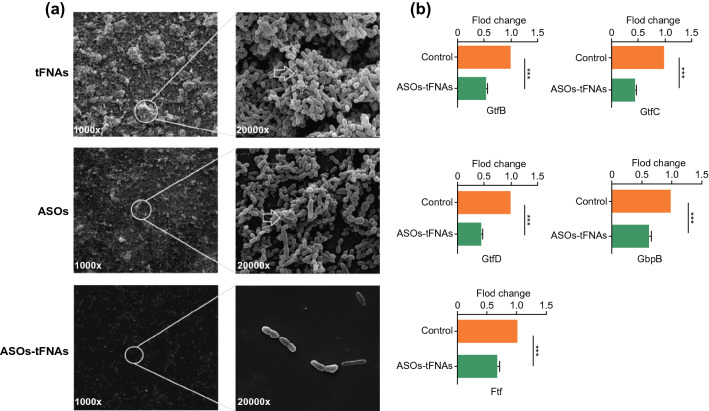
Cellular and molecular mechanisms responsible for inhibiting biofilm formation upon ASOs-tFNAs targeting. **a** Representative SEM images showing the architecture of biofilms following 24 h of incubation with tFNAs, ASOs, or ASOs-tFNAs. Images were taken at × 1000 and × 20,000 magnification, **b** expression of target genes as determined by quantitative RT-PCR. 16S rRNA was used as control. ***P < 0.001

The effect of ASOs-tFNAs on biofilm formation was confirmed also by SEM (Fig. 5a). Consistent with confocal imaging results, the biofilms treated with 750 nM ASOs-tFNAs showed a significant reduction in EPS, resulting in a spongier and more porous structure compared with tFNAs and ASOs treatments (white arrows). In addition, *S. mutans* did not show morphological abnormalities, such as damaged cell walls or swelling, demonstrating that ASOs-tFNAs had no significant bactericidal effect.

### ASOs-tFNAs Inhibit Biofilm Formation

Finally, we sought to determine the molecular mechanism by which ASOs-tFNAs inhibited bacterial biofilm formation. After culturing *S. mutans* in the presence of different concentrations of ASOs-tFNAs for 24 h, total RNA was extracted from equal amounts of bacterial cells and gene expression was analyzed by quantitative RT-PCR. As shown in Fig. 5b, ASOs-tFNAs inhibited expression of the *gtfBCD, gbpB,* and *ftf* genes. This finding demonstrated that the delivery system could simultaneously reduce the expression of all targeted genes. Moreover, expression of target genes was reduced compared to 16S rRNA, indicating that ASOs-tFNAs targeting was specific for the intended genes.

There are many methods for treating chronic infections associated with biofilms, but none of them is 100% effective. During biofilm formation, the EPS enters deep into tissue structures, thus hindering its complete removal. Moreover, the formation of biofilms allows bacteria to acquire many resistance mechanisms against commonly used antibiotics. Critically, the multi-targeted antisense oligonucleotide delivery system proposed here targets a variety of genes implicated in the early stages of biofilm formation, effectively limiting its occurrence. Based on our results, an ASO-tFNAs concentration of 750 nM achieves substantial EPS synthesis inhibition and, hence, reduction in biofilm formation and virulence during its early stages.

## Conclusions

**Fig. 6:**
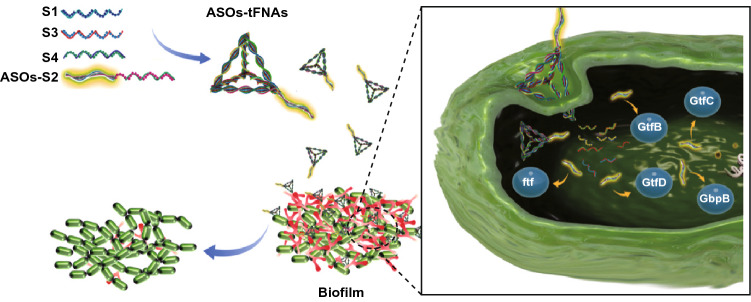
Schematic representation of how tFNAs deliver ASOs to inhibit the formation of bacterial biofilms by targeting genes related to EPS synthesis

We report constructing a novel and effective tFNA system for delivering ASOs. We verified the stability of the delivery system and demonstrated that ASOs-tFNAs could penetrate the cell wall of *S. mutans*. Besides carrying antisense oligonucleotides to specific genes and thus downregulating their expression, ASOs-tFNAs can be designed to target multiple genes, which critically improves their inhibitory action (Fig. 6) [42]. By inhibiting the early stages of biofilm formation, this strategy allows the treatment of chronic biofilm-mediated infection through subsequent early debridement or by improving the effect of antibiotics, both of which are otherwise powerless in already formed biofilms. Our results have validated the application of tFNAs as drug delivery systems in *S. mutans*; however, more types of biofilms and bacterial strains should be studied. We anticipate that tFNAs delivery systems can have a significant potential for the systemic inhibition of bacterial biofilms.
